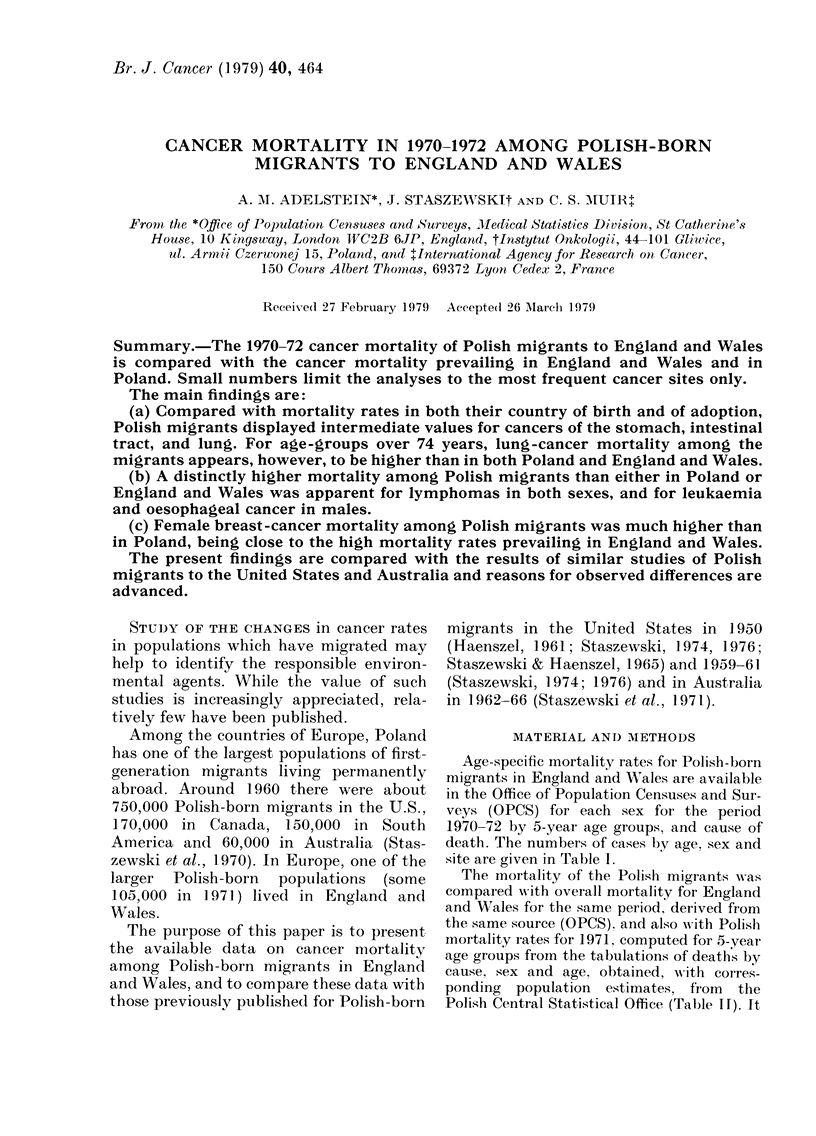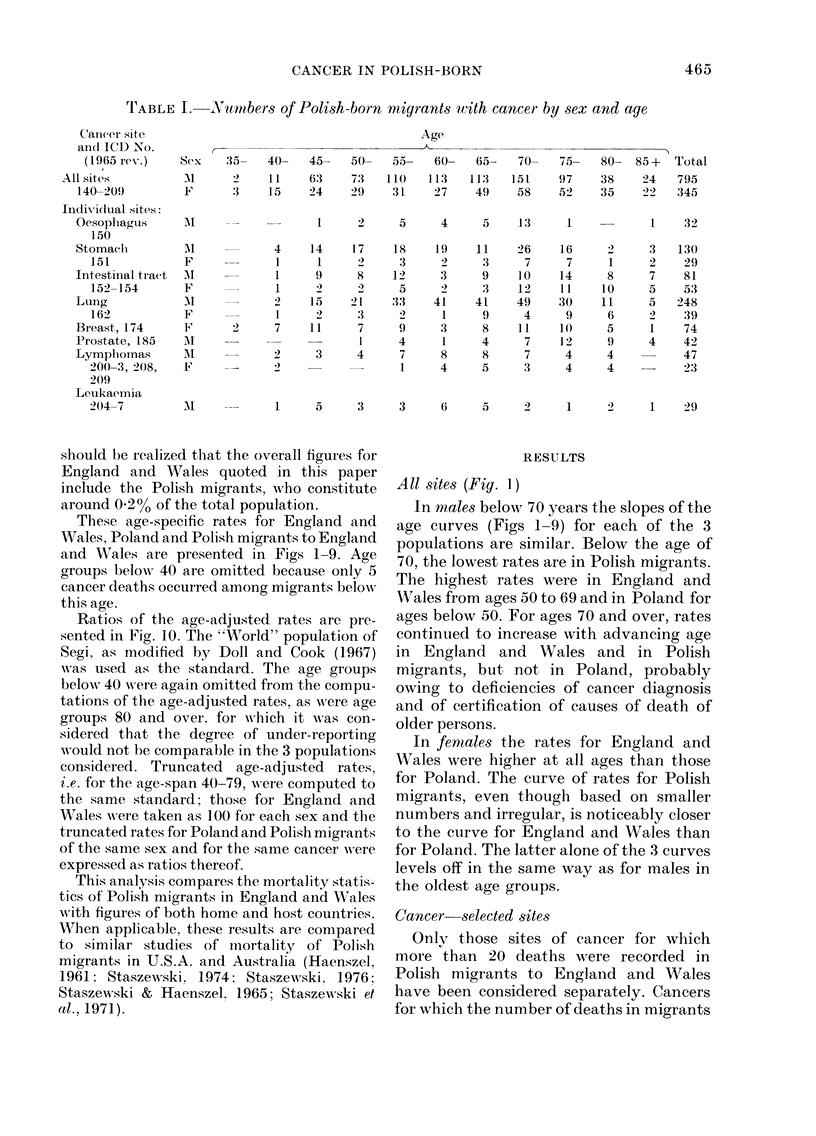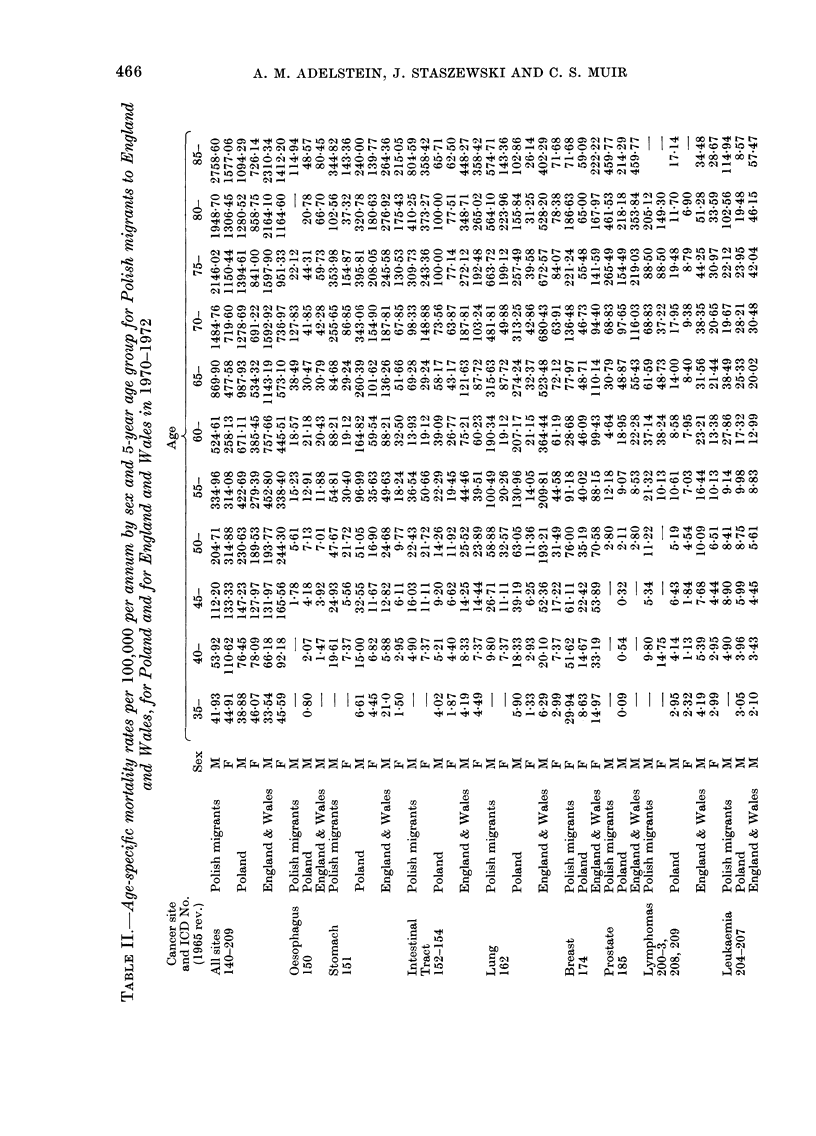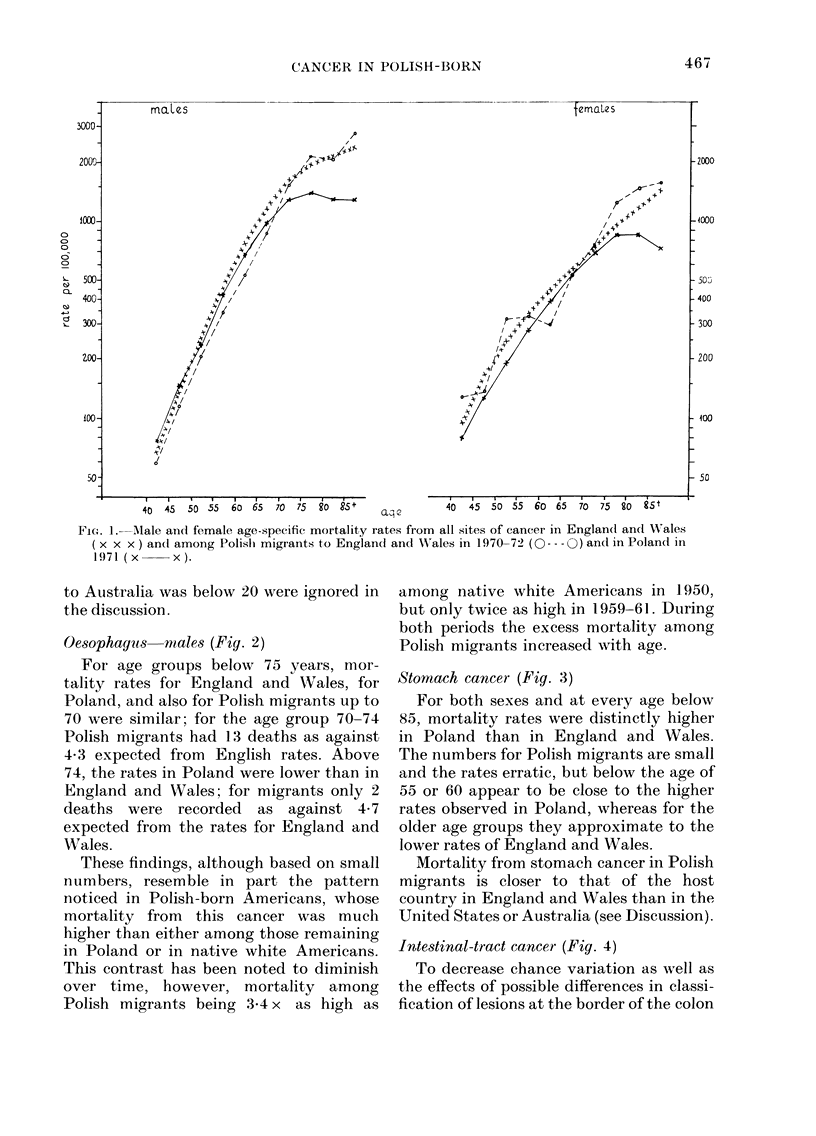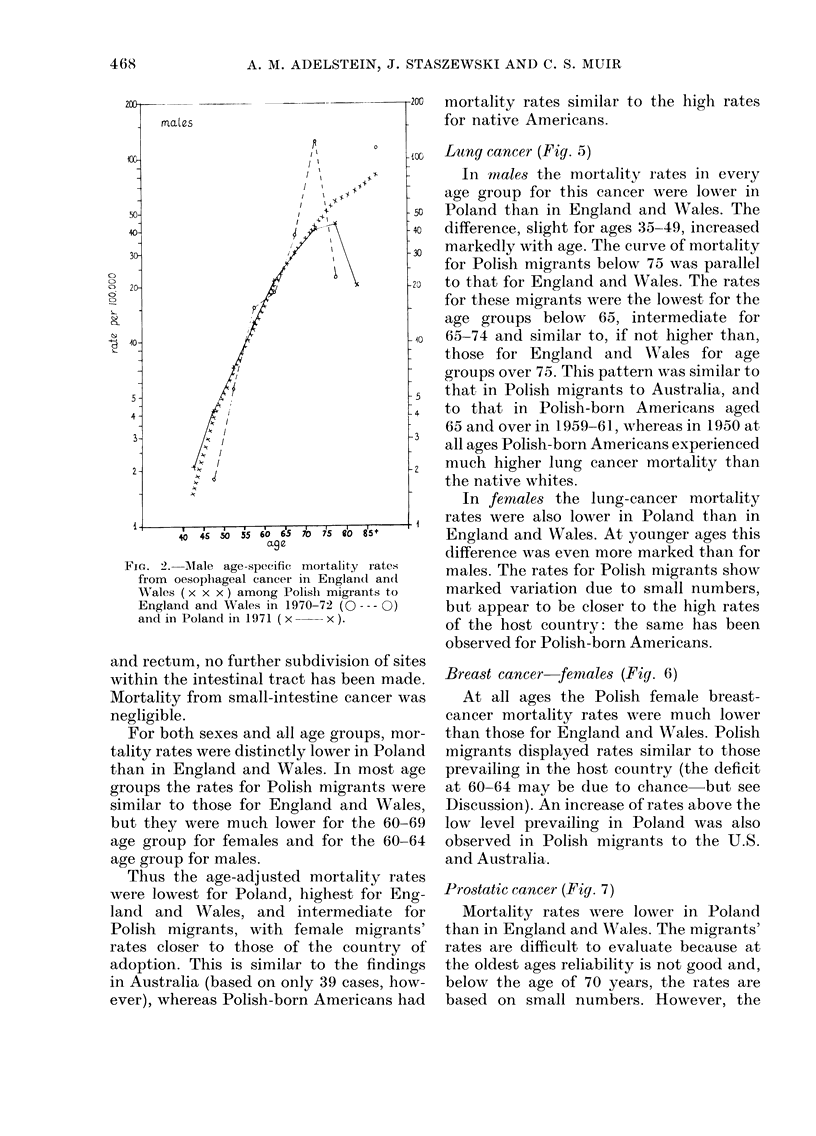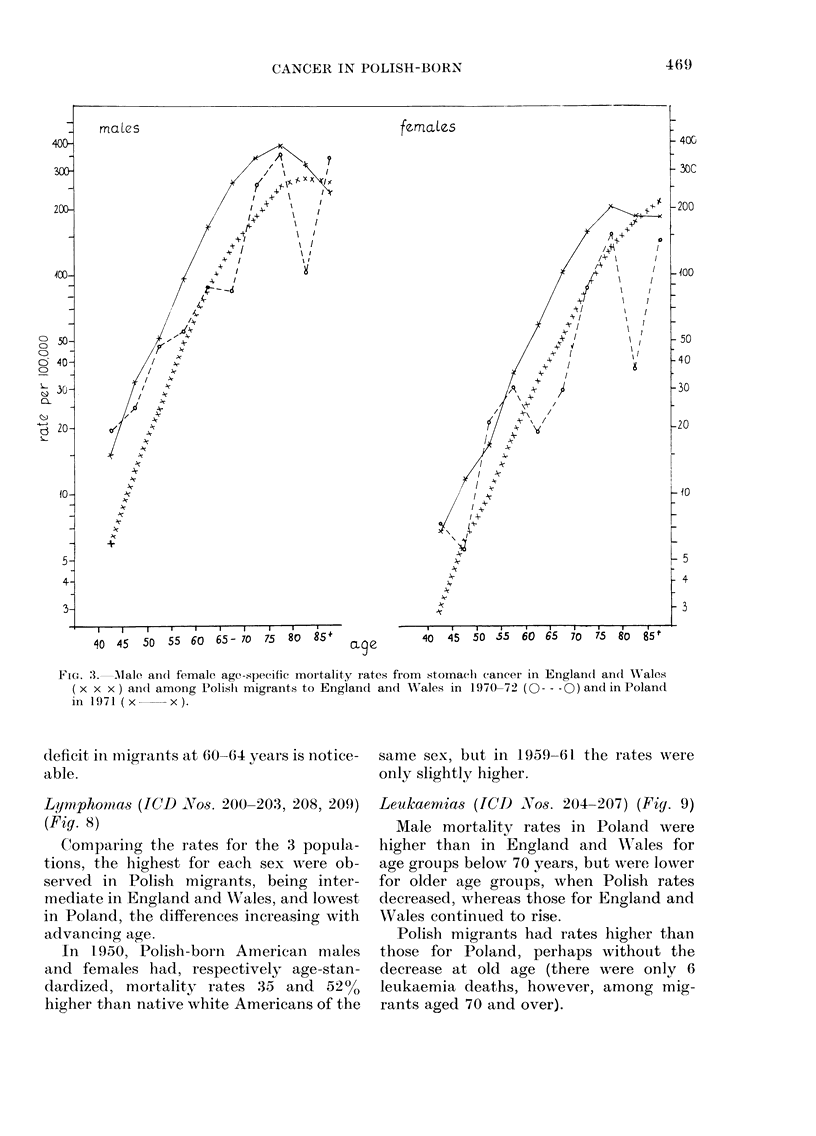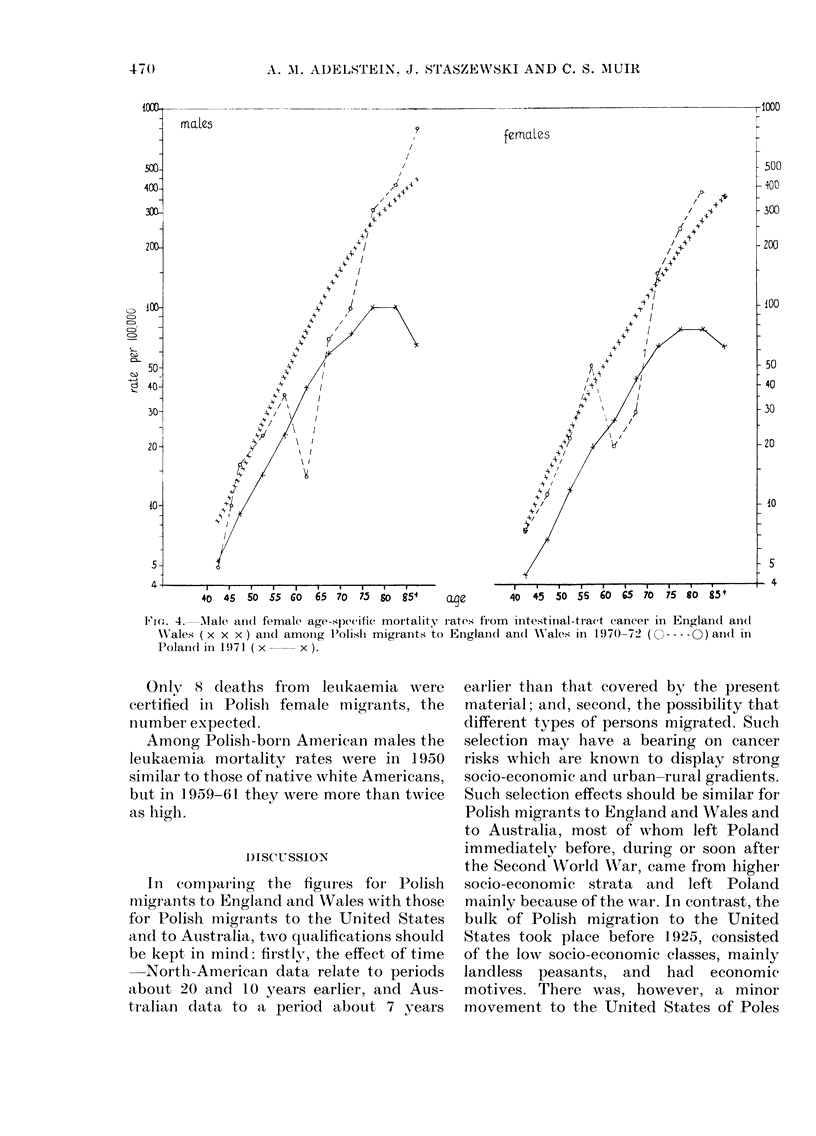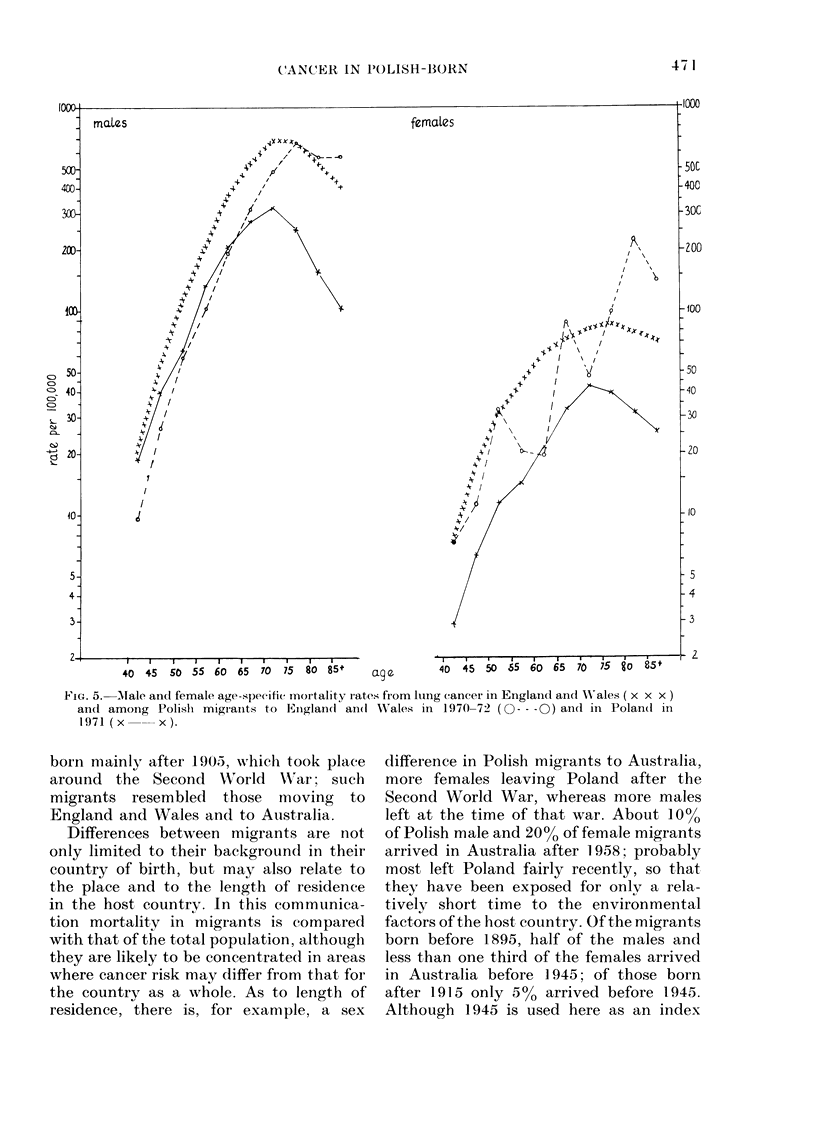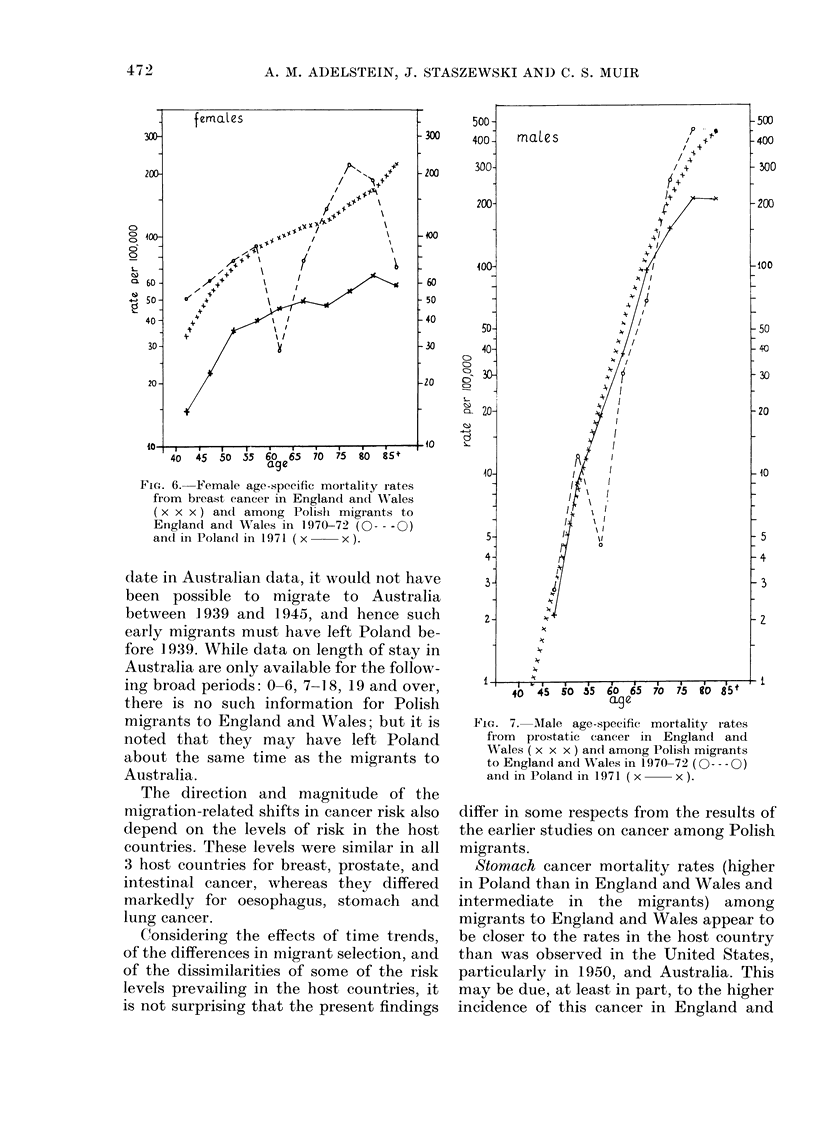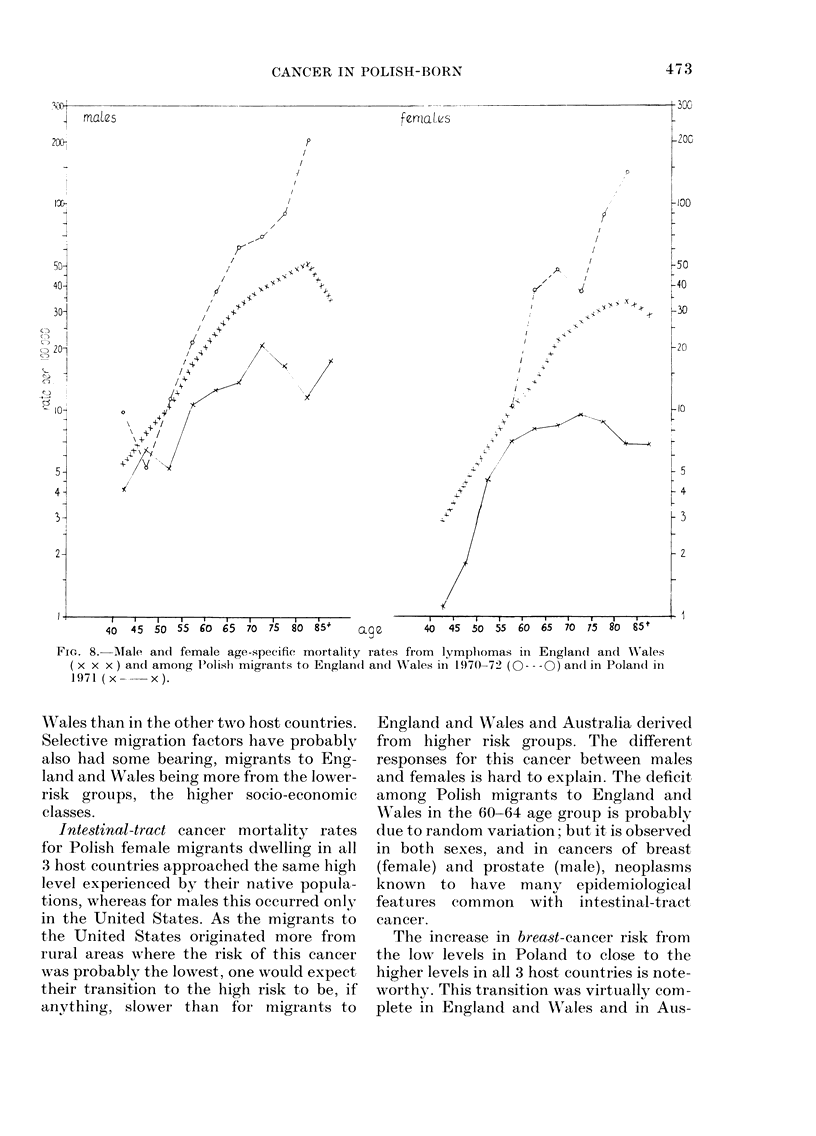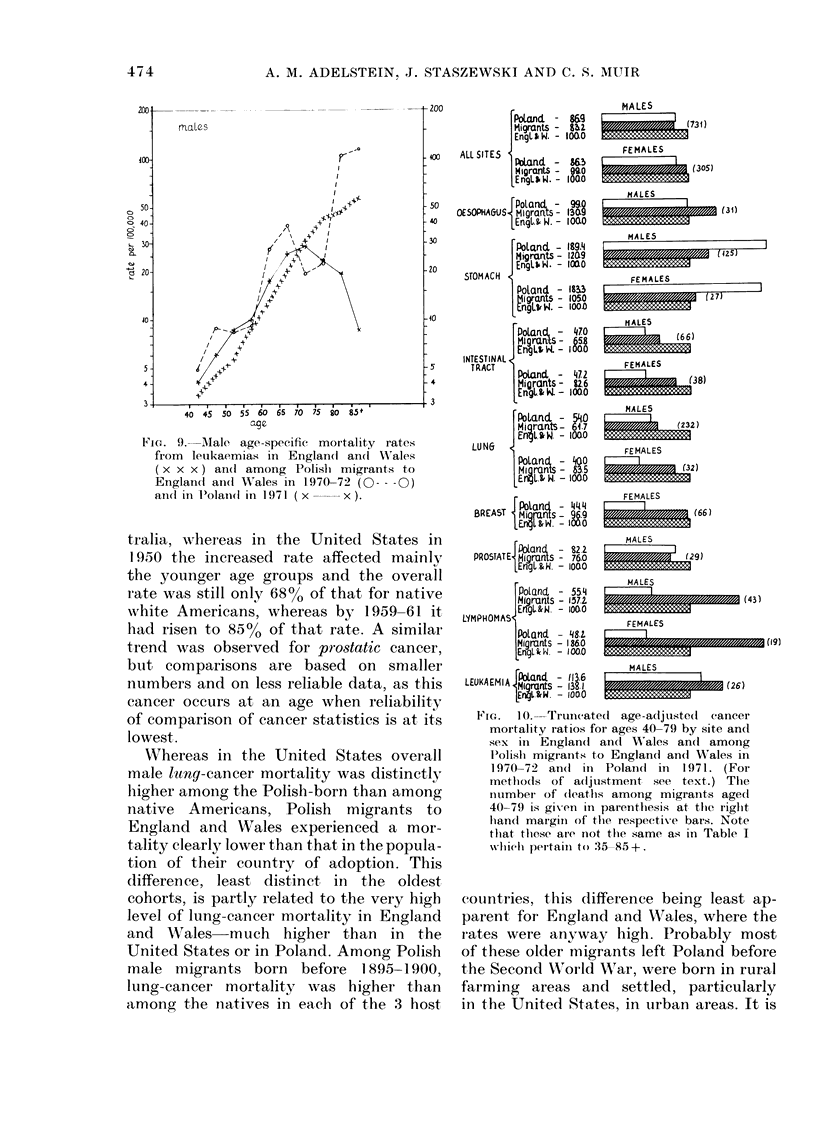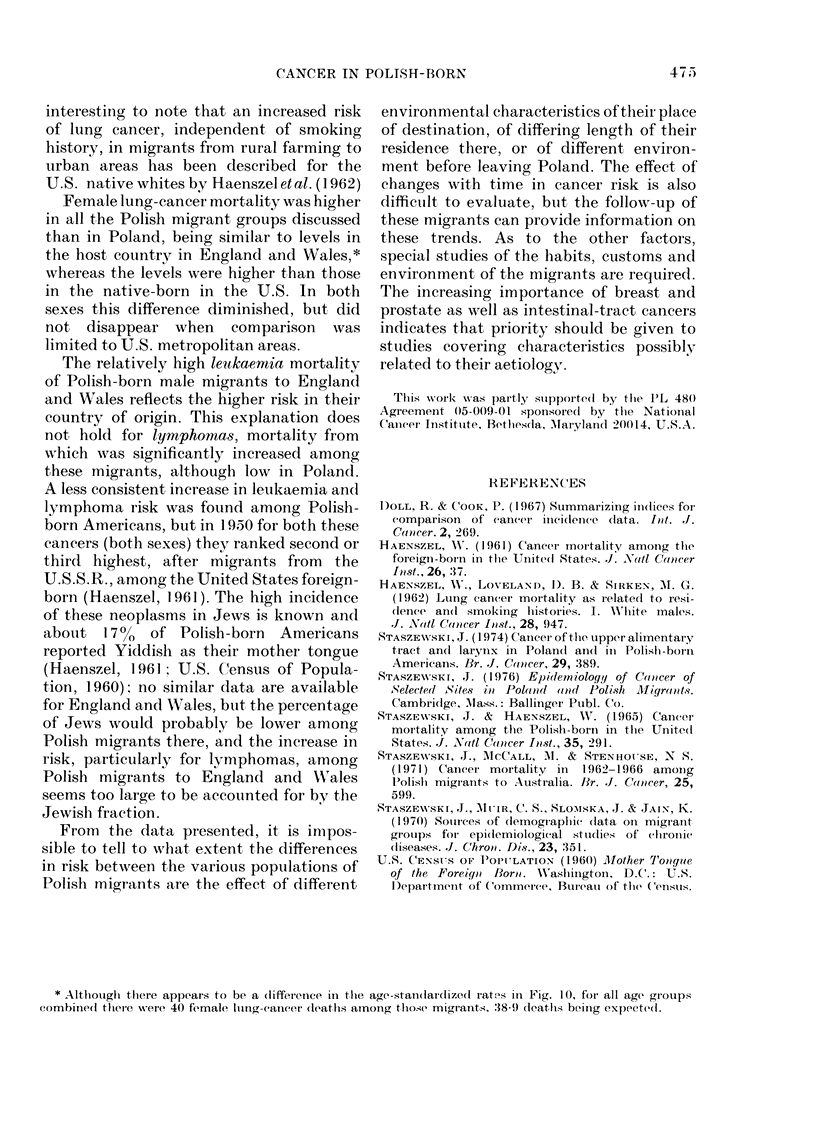# Cancer mortality in 1970-1972 among Polish-born migrants to England and Wales.

**DOI:** 10.1038/bjc.1979.202

**Published:** 1979-09

**Authors:** A. M. Adelstein, J. Staszewski, C. S. Muir

## Abstract

The 1970-72 cancer mortality of Polish migrants to England and Wales is compared with the cancer mortality prevailing in England and Wales and in Poland. Small numbers limit the analyses to the most frequent cancer sites only. The main findings are: (a) Compared with mortality rates in both their country of birth and of adoption, Polish migrants displayed intermediate values for cancers of the stomach, intestinal tract, and lung. For age-groups over 74 years, lung-cancer mortality among the migrants appears, however, to be higher than in both Poland and England and Wales. (b) A distinctly higher mortality among Polish migrants than either in Poland or England and Wales was apparent for lymphomas in both sexes, and for leukaemia and oesophageal cancer in males. (c) Female breast-cancer mortality among Polish migrants was much higher than in Poland, being close to the high mortality rates prevailing in England and Wales. The present findings are compared with the results of similar studies of Polish migrants to the United States and Australia and reasons for observed differences are advanced.


*Br. J. Cancer* (1979) **40**, 464

### CANCER MORTALITY IN 1970–1972 AMONG POLISH-BORN MIGRANTS TO ENGLAND AND WALES

A. M. ADELSTEIN*, J. STASZEWSKI† AND C. S. MUIR‡

*From the *Office of Population Censuses and Surveys, Medical Statistics Division, St Catherine's House,* 10 *Kingsway, London WC2B 6JP, England, †Instytut Onkologii,* 44–101 *Gliwice, ul. Armii Czerwonej* 15, *Poland, and ‡International Agency for Research on Cancer,* 150 *Cours Albert Thomas,* 69372 *Lyon Cedex* 2, *France*

Received 27 February 1979 Accepted 26 March 1979

**Summary.—The 1970–72 cancer mortality of Polish migrants to England and Wales is compared with the cancer mortality prevailing in England and Wales and in Poland. Small numbers limit the analyses to the most frequent cancer sites only.**

**The main findings are:**

**(a) Compared with mortality rates in both their country of birth and of adoption, Polish migrants displayed intermediate values for cancers of the stomach, intestinal tract, and lung. For age-groups over 74 years, lung-cancer mortality among the migrants appears, however, to be higher than in both Poland and England and Wales.**

**(b) A distinctly higher mortality among Polish migrants than either in Poland or England and Wales was apparent for lymphomas in both sexes, and for leukaemia and oesophageal cancer in males.**

**(c) Female breast-cancer mortality among Polish migrants was much higher than in Poland, being close to the high mortality rates prevailing in England and Wales.**

**The present findings are compared with the results of similar studies of Polish migrants to the United States and Australia and reasons for observed differences are advanced.**

STUDY OF THE CHANGES in cancer rates in populations which have migrated may help to identify the responsible environmental agents. While the value of such studies is increasingly appreciated, relatively few have been published.

Among the countries of Europe, Poland has one of the largest populations of first-generation migrants living permanently abroad. Around 1960 there were about 750,000 Polish-born migrants in the U.S., 170,000 in Canada, 150,000 in South America and 60,000 in Australia (Staszewski *et al.*, 1970). In Europe, one of the larger Polish-born populations (some 105,000 in 1971) lived in England and Wales.

The purpose of this paper is to present the available data on cancer mortality among Polish-born migrants in England and Wales, and to compare these data with those previously published for Polish-born migrants in the United States in 1950 (Haenszel, 1961; Staszewski, 1974, 1976; Staszewski & Haenszel, 1965) and 1959–61 (Staszewski, 1974; 1976) and in Australia in 1962–66 (Staszewski *et al.*, 1971).

### MATERIAL AND METHODS

Age-specific mortality rates for Polish-born migrants in England and Wales are available in the Office of Population Censuses and Surveys (OPCS) for each sex for the period 1970–72 by 5-year age groups, and cause of death. The numbers of cases by age, sex and site are given in Table I.

The mortality of the Polish migrants was compared with overall mortality for England and Wales for the same period, derived from the same source (OPCS), and also with Polish mortality rates for 1971, computed for 5-year age groups from the tabulations of deaths by cause, sex and age, obtained, with corresponding population estimates, from the Polish Central Statistical Office (Table II). It

TABLE I.—*Numbers of Polish-born migrants with cancer by sex and age*

| Cancer site and ICD No. (1965 rev.) | Sex | Age 35– | 40– | 45– | 50– | 55– | 60– | 65– | 70– | 75– | 80– | 85+ | Total |
|---|---|---|---|---|---|---|---|---|---|---|---|---|---|
| All sites | M | 2 | 11 | 63 | 73 | 110 | 113 | 113 | 151 | 97 | 38 | 24 | 795 |
| 140–209 | F | 3 | 15 | 24 | 29 | 31 | 27 | 49 | 58 | 52 | 35 | 22 | 345 |
| Individual sites: | | | | | | | | | | | | | |
| Oesophagus 150 | M | — | — | 1 | 2 | 5 | 4 | 5 | 13 | 1 | — | 1 | 32 |
| Stomach | M | — | 4 | 14 | 17 | 18 | 19 | 11 | 26 | 16 | 2 | 3 | 130 |
| 151 | F | — | 1 | 1 | 2 | 3 | 2 | 3 | 7 | 7 | 1 | 2 | 29 |
| Intestinal tract | M | — | 1 | 9 | 8 | 12 | 3 | 9 | 10 | 14 | 8 | 7 | 81 |
| 152–154 | F | — | 1 | 2 | 2 | 5 | 2 | 3 | 12 | 11 | 10 | 5 | 53 |
| Lung | M | — | 2 | 15 | 21 | 33 | 41 | 41 | 49 | 30 | 11 | 5 | 248 |
| 162 | F | — | 1 | 2 | 3 | 2 | 1 | 9 | 4 | 9 | 6 | 2 | 39 |
| Breast, 174 | F | 2 | 7 | 11 | 7 | 9 | 3 | 8 | 11 | 10 | 5 | 1 | 74 |
| Prostate, 185 | M | — | — | — | 1 | 4 | 1 | 4 | 7 | 12 | 9 | 4 | 42 |
| Lymphomas | M | — | 2 | 3 | 4 | 7 | 8 | 8 | 7 | 4 | 4 | — | 47 |
| 200–3, 208, 209 | F | — | 2 | — | — | 1 | 4 | 5 | 3 | 4 | 4 | — | 23 |
| Leukaemia 204–7 | M | — | 1 | 5 | 3 | 3 | 6 | 5 | 2 | 1 | 2 | 1 | 29 |

should be realized that the overall figures for England and Wales quoted in this paper include the Polish migrants, who constitute around 0·2% of the total population.

These age-specific rates for England and Wales, Poland and Polish migrants to England and Wales are presented in Figs 1–9. Age groups below 40 are omitted because only 5 cancer deaths occurred among migrants below this age.

Ratios of the age-adjusted rates are presented in Fig. 10. The "World" population of Segi, as modified by Doll and Cook (1967) was used as the standard. The age groups below 40 were again omitted from the computations of the age-adjusted rates, as were age groups 80 and over, for which it was considered that the degree of under-reporting would not be comparable in the 3 populations considered. Truncated age-adjusted rates, *i.e.* for the age-span 40–79, were computed to the same standard; those for England and Wales were taken as 100 for each sex and the truncated rates for Poland and Polish migrants of the same sex and for the same cancer were expressed as ratios thereof.

This analysis compares the mortality statistics of Polish migrants in England and Wales with figures of both home and host countries. When applicable, these results are compared to similar studies of mortality of Polish migrants in U.S.A. and Australia (Haenszel, 1961; Staszewski, 1974; Staszewski, 1976; Staszewski & Haenszel, 1965; Staszewski *et al.*, 1971).

### RESULTS

### *All sites* (*Fig.* 1)

In *males* below 70 years the slopes of the age curves (Figs 1–9) for each of the 3 populations are similar. Below the age of 70, the lowest rates are in Polish migrants. The highest rates were in England and Wales from ages 50 to 69 and in Poland for ages below 50. For ages 70 and over, rates continued to increase with advancing age in England and Wales and in Polish migrants, but not in Poland, probably owing to deficiencies of cancer diagnosis and of certification of causes of death of older persons.

In *females* the rates for England and Wales were higher at all ages than those for Poland. The curve of rates for Polish migrants, even though based on smaller numbers and irregular, is noticeably closer to the curve for England and Wales than for Poland. The latter alone of the 3 curves levels off in the same way as for males in the oldest age groups.

### *Cancer—selected sites*

Only those sites of cancer for which more than 20 deaths were recorded in Polish migrants to England and Wales have been considered separately. Cancers for which the number of deaths in migrants

TABLE II.—*Age-specific mortality rates per* 100,000 *per annum by sex and* 5*-year age group for Polish migrants to England and Wales, for Poland and for England and Wales in* 1970–1972

| Cancer site and ICD No. (1965 rev.) | | Sex | Age 35– | 40– | 45– | 50– | 55– | 60– | 65– | 70– | 75– | 80– | 85– |
|---|---|---|---|---|---|---|---|---|---|---|---|---|---|
| All sites | Polish migrants | M | 41·93 | 53·92 | 112·20 | 204·71 | 334·96 | 524·61 | 869·90 | 1484·76 | 2146·02 | 1948·70 | 2758·60 |
| 140–209 | | F | 44·91 | 110·62 | 133·33 | 314·88 | 314·08 | 258·13 | 477·58 | 719·60 | 1150·44 | 1306·45 | 1577·06 |
| | Poland | M | 38·88 | 76·45 | 147·23 | 230·63 | 422·69 | 671·11 | 987·93 | 1278·69 | 1394·61 | 1280·52 | 1094·29 |
| | | F | 46·07 | 78·09 | 127·97 | 189·53 | 279·39 | 385·45 | 534·32 | 691·22 | 841·00 | 858·75 | 726·14 |
| | England & Wales | M | 33·54 | 66·18 | 131·97 | 193·77 | 452·80 | 757·66 | 1143·19 | 1592·92 | 1597·90 | 2164·10 | 2310·34 |
| | | F | 45·59 | 92·18 | 165·56 | 244·30 | 338·40 | 445·51 | 573·10 | 736·97 | 951·33 | 1164·60 | 1412·20 |
| Oesophagus | Polish migrants | M | — | — | 1·78 | 5·61 | 15·23 | 18·57 | 38·49 | 127·83 | 22·12 | — | 114·94 |
| 150 | Poland | M | 0·80 | 2·07 | 4·18 | 7·13 | 12·91 | 21·18 | 30·47 | 41·85 | 44·31 | 20·78 | 48·57 |
| | England & Wales | M | — | 1·47 | 3·92 | 7·01 | 11·88 | 20·43 | 30·79 | 42·28 | 59·73 | 66·70 | 80·45 |
| Stomach | Polish migrants | M | — | 19·61 | 24·93 | 47·67 | 54·81 | 88·21 | 84·68 | 255·65 | 353·98 | 102·56 | 344·82 |
| 151 | | F | — | 7·37 | 5·56 | 21·72 | 30·40 | 19·12 | 29·24 | 86·85 | 154·87 | 37·32 | 143·36 |
| | Poland | M | 6·61 | 15·00 | 32·55 | 51·05 | 96·99 | 164·82 | 260·39 | 343·06 | 395·81 | 320·78 | 240·00 |
| | | F | 4·45 | 6·82 | 11·67 | 16·90 | 35·63 | 59·54 | 101·62 | 154·90 | 208·05 | 180·63 | 139·77 |
| | England & Wales | M | 21·0 | 5·88 | 12·82 | 24·68 | 49·63 | 88·21 | 136·26 | 187·81 | 245·58 | 276·92 | 264·36 |
| | | F | 1·50 | 2·95 | 6·11 | 9·77 | 18·24 | 32·50 | 51·66 | 67·85 | 130·53 | 175·43 | 215·05 |
| Intestinal | Polish migrants | M | — | 4·90 | 16·03 | 22·43 | 36·54 | 13·93 | 69·28 | 98·33 | 309·73 | 410·25 | 804·59 |
| Tract | | F | — | 7·37 | 11·11 | 21·72 | 50·66 | 19·12 | 29·24 | 148·88 | 243·36 | 373·27 | 358·42 |
| 152–154 | Poland | M | 4·02 | 5·21 | 9·20 | 14·26 | 22·29 | 39·09 | 58·17 | 73·56 | 100·00 | 100·00 | 65·71 |
| | | F | 1·87 | 4·40 | 6·62 | 11·92 | 19·45 | 26·77 | 43·17 | 63·87 | 77·14 | 77·51 | 62·50 |
| | England & Wales | M | 4·19 | 8·33 | 14·25 | 25·52 | 44·46 | 75·21 | 121·63 | 187·81 | 272·12 | 348·71 | 448·27 |
| | | F | 4·49 | 7·37 | 14·44 | 23·89 | 39·51 | 60·23 | 87·72 | 103·24 | 192·48 | 265·02 | 358·42 |
| Lung | Polish migrants | M | — | 9·80 | 26·71 | 58·88 | 100·49 | 190·34 | 315·63 | 481·81 | 663·72 | 564·10 | 574·71 |
| 162 | | F | — | 7·37 | 11·11 | 32·57 | 20·26 | 19·12 | 87·72 | 49·88 | 199·12 | 223·96 | 143·36 |
| | Poland | M | 5·90 | 18·33 | 39·19 | 63·05 | 130·96 | 207·17 | 274·24 | 313·25 | 257·49 | 155·84 | 102·86 |
| | | F | 1·33 | 2·93 | 6·25 | 11·36 | 14·05 | 21·15 | 32·37 | 42·86 | 39·58 | 31·25 | 26·14 |
| | England & Wales | M | 6·29 | 20·10 | 52·36 | 193·21 | 209·81 | 364·44 | 523·48 | 680·43 | 672·57 | 528·20 | 402·29 |
| | | F | 2·99 | 7·37 | 17·22 | 31·49 | 44·58 | 61·19 | 72·12 | 63·91 | 84·07 | 78·38 | 71·68 |
| Breast | Polish migrants | F | 29·94 | 51·62 | 61·11 | 76·00 | 91·18 | 28·68 | 77·97 | 136·48 | 221·24 | 186·63 | 71·68 |
| 174 | Poland | F | 8·63 | 14·67 | 22·42 | 35·19 | 40·02 | 46·09 | 48·71 | 46·73 | 55·48 | 65·00 | 59·09 |
| | England & Wales | F | 14·97 | 33·19 | 53·89 | 70·58 | 88·15 | 99·43 | 110·14 | 94·40 | 141·59 | 167·97 | 222·22 |
| Prostate | Polish migrants | M | — | — | — | 2·80 | 12·18 | 4·64 | 30·79 | 68·83 | 265·49 | 461·53 | 459·77 |
| 185 | Poland | M | 0·09 | 0·54 | 0·32 | 2·11 | 9·07 | 18·95 | 48·87 | 97·65 | 154·49 | 218·18 | 214·29 |
| | England & Wales | M | — | — | — | 2·80 | 8·53 | 22·28 | 55·43 | 116·03 | 219·03 | 353·84 | 459·77 |
| Lymphomas | Polish migrants | M | — | 9·80 | 5·34 | 11·22 | 21·32 | 37·14 | 61·59 | 68·83 | 88·50 | 205·12 | — |
| 200–3, | | F | — | 14·75 | — | — | 10·13 | 38·24 | 48·73 | 37·22 | 88·50 | 149·30 | — |
| 208, 209 | Poland | M | 2·95 | 4·14 | 6·43 | 5·19 | 10·61 | 8·58 | 14·00 | 17·95 | 19·48 | 11·70 | 17·14 |
| | | F | 2·32 | 1·13 | 1·84 | 4·54 | 7·03 | 7·95 | 8·40 | 9·38 | 8·79 | 6·90 | — |
| | England & Wales | M | 4·19 | 5·39 | 7·68 | 10·09 | 16·44 | 23·21 | 31·56 | 38·35 | 44·25 | 51·28 | 34·48 |
| | | F | 2·99 | 2·95 | 4·44 | 6·51 | 10·13 | 13·38 | 21·44 | 20·65 | 30·97 | 33·59 | 28·67 |
| Leukaemia | Polish migrants | M | — | 4·90 | 8·90 | 8·41 | 9·14 | 27·86 | 38·49 | 19·67 | 22·12 | 102·56 | 114·94 |
| 204–207 | Poland | M | 3·05 | 3·96 | 5·99 | 8·75 | 9·98 | 17·32 | 25·33 | 28·21 | 23·95 | 19·48 | 8·57 |
| | England & Wales | M | 2·10 | 3·43 | 4·45 | 5·61 | 8·83 | 12·99 | 20·02 | 30·48 | 42·04 | 46·15 | 57·47 |

FIG. 1.—Male and female age-specific mortality rates from all sites of cancer in England and Wales (× × ×) and among Polish migrants to England and Wales in 1970–72 (O- - -O) and in Poland in 1971 (×——×).

to Australia was below 20 were ignored in the discussion.

*Oesophagus—males* (*Fig.* 2)

For age groups below 75 years, mortality rates for England and Wales, for Poland, and also for Polish migrants up to 70 were similar; for the age group 70–74 Polish migrants had 13 deaths as against 4·3 expected from English rates. Above 74, the rates in Poland were lower than in England and Wales; for migrants only 2 deaths were recorded as against 4·7 expected from the rates for England and Wales.

These findings, although based on small numbers, resemble in part the pattern noticed in Polish-born Americans, whose mortality from this cancer was much higher than either among those remaining in Poland or in native white Americans. This contrast has been noted to diminish over time, however, mortality among Polish migrants being 3·4 × as high as among native white Americans in 1950, but only twice as high in 1959–61. During both periods the excess mortality among Polish migrants increased with age.

*Stomach cancer* (*Fig.* 3)

For both sexes and at every age below 85, mortality rates were distinctly higher in Poland than in England and Wales. The numbers for Polish migrants are small and the rates erratic, but below the age of 55 or 60 appear to be close to the higher rates observed in Poland, whereas for the older age groups they approximate to the lower rates of England and Wales.

Mortality from stomach cancer in Polish migrants is closer to that of the host country in England and Wales than in the United States or Australia (see Discussion).

*Intestinal-tract cancer* (*Fig.* 4)

To decrease chance variation as well as the effects of possible differences in classification of lesions at the border of the colon

FIG. 2.—Male age-specific mortality rates from oesophageal cancer in England and Wales (× × ×) among Polish migrants to England and Wales in 1970–72 (○ - - - ○) and in Poland in 1971 (× —— ×).

and rectum, no further subdivision of sites within the intestinal tract has been made. Mortality from small-intestine cancer was negligible.

For both sexes and all age groups, mortality rates were distinctly lower in Poland than in England and Wales. In most age groups the rates for Polish migrants were similar to those for England and Wales, but they were much lower for the 60–69 age group for females and for the 60–64 age group for males.

Thus the age-adjusted mortality rates were lowest for Poland, highest for England and Wales, and intermediate for Polish migrants, with female migrants' rates closer to those of the country of adoption. This is similar to the findings in Australia (based on only 39 cases, however), whereas Polish-born Americans had mortality rates similar to the high rates for native Americans.

*Lung cancer* (*Fig.* 5)

In *males* the mortality rates in every age group for this cancer were lower in Poland than in England and Wales. The difference, slight for ages 35–49, increased markedly with age. The curve of mortality for Polish migrants below 75 was parallel to that for England and Wales. The rates for these migrants were the lowest for the age groups below 65, intermediate for 65–74 and similar to, if not higher than, those for England and Wales for age groups over 75. This pattern was similar to that in Polish migrants to Australia, and to that in Polish-born Americans aged 65 and over in 1959–61, whereas in 1950 at all ages Polish-born Americans experienced much higher lung cancer mortality than the native whites.

In *females* the lung-cancer mortality rates were also lower in Poland than in England and Wales. At younger ages this difference was even more marked than for males. The rates for Polish migrants show marked variation due to small numbers, but appear to be closer to the high rates of the host country: the same has been observed for Polish-born Americans.

*Breast cancer—females* (*Fig.* 6)

At all ages the Polish female breast-cancer mortality rates were much lower than those for England and Wales. Polish migrants displayed rates similar to those prevailing in the host country (the deficit at 60–64 may be due to chance—but see Discussion). An increase of rates above the low level prevailing in Poland was also observed in Polish migrants to the U.S. and Australia.

*Prostatic cancer* (*Fig.* 7)

Mortality rates were lower in Poland than in England and Wales. The migrants' rates are difficult to evaluate because at the oldest ages reliability is not good and, below the age of 70 years, the rates are based on small numbers. However, the

FIG. 3.—Male and female age-specific mortality rates from stomach cancer in England and Wales (× × ×) and among Polish migrants to England and Wales in 1970–72 (○- - -○) and in Poland in 1971 (×——×).

deficit in migrants at 60–64 years is noticeable.

*Lymphomas* (*ICD Nos.* 200–203, 208, 209) (*Fig.* 8)

Comparing the rates for the 3 populations, the highest for each sex were observed in Polish migrants, being intermediate in England and Wales, and lowest in Poland, the differences increasing with advancing age.

In 1950, Polish-born American males and females had, respectively age-standardized, mortality rates 35 and 52% higher than native white Americans of the same sex, but in 1959–61 the rates were only slightly higher.

*Leukaemias* (*ICD Nos.* 204–207) (*Fig.* 9)

Male mortality rates in Poland were higher than in England and Wales for age groups below 70 years, but were lower for older age groups, when Polish rates decreased, whereas those for England and Wales continued to rise.

Polish migrants had rates higher than those for Poland, perhaps without the decrease at old age (there were only 6 leukaemia deaths, however, among migrants aged 70 and over).

FIG. 4.—Male and female age-specific mortality rates from intestinal-tract cancer in England and Wales ( x x x ) and among Polish migrants to England and Wales in 1970–72 (○- - - -○) and in Poland in 1971 ( x —— x ).

Only 8 deaths from leukaemia were certified in Polish female migrants, the number expected.

Among Polish-born American males the leukaemia mortality rates were in 1950 similar to those of native white Americans, but in 1959–61 they were more than twice as high.

### DISCUSSION

In comparing the figures for Polish migrants to England and Wales with those for Polish migrants to the United States and to Australia, two qualifications should be kept in mind: firstly, the effect of time—North-American data relate to periods about 20 and 10 years earlier, and Australian data to a period about 7 years earlier than that covered by the present material; and, second, the possibility that different types of persons migrated. Such selection may have a bearing on cancer risks which are known to display strong socio-economic and urban–rural gradients. Such selection effects should be similar for Polish migrants to England and Wales and to Australia, most of whom left Poland immediately before, during or soon after the Second World War, came from higher socio-economic strata and left Poland mainly because of the war. In contrast, the bulk of Polish migration to the United States took place before 1925, consisted of the low socio-economic classes, mainly landless peasants, and had economic motives. There was, however, a minor movement to the United States of Poles

FIG. 5.—Male and female age-specific mortality rates from lung cancer in England and Wales ( x x x ) and among Polish migrants to England and Wales in 1970–72 (O- - -O) and in Poland in 1971 ( x —— x ).

born mainly after 1905, which took place around the Second World War; such migrants resembled those moving to England and Wales and to Australia.

Differences between migrants are not only limited to their background in their country of birth, but may also relate to the place and to the length of residence in the host country. In this communication mortality in migrants is compared with that of the total population, although they are likely to be concentrated in areas where cancer risk may differ from that for the country as a whole. As to length of residence, there is, for example, a sex difference in Polish migrants to Australia, more females leaving Poland after the Second World War, whereas more males left at the time of that war. About 10% of Polish male and 20% of female migrants arrived in Australia after 1958; probably most left Poland fairly recently, so that they have been exposed for only a relatively short time to the environmental factors of the host country. Of the migrants born before 1895, half of the males and less than one third of the females arrived in Australia before 1945; of those born after 1915 only 5% arrived before 1945. Although 1945 is used here as an index

FIG. 6.—Female age-specific mortality rates from breast cancer in England and Wales (× × ×) and among Polish migrants to England and Wales in 1970–72 (○- - -○) and in Poland in 1971 (×——×).

FIG. 7.—Male age-specific mortality rates from prostatic cancer in England and Wales (× × ×) and among Polish migrants to England and Wales in 1970–72 (○- - -○) and in Poland in 1971 (×——×).

date in Australian data, it would not have been possible to migrate to Australia between 1939 and 1945, and hence such early migrants must have left Poland before 1939. While data on length of stay in Australia are only available for the following broad periods: 0–6, 7–18, 19 and over, there is no such information for Polish migrants to England and Wales; but it is noted that they may have left Poland about the same time as the migrants to Australia.

The direction and magnitude of the migration-related shifts in cancer risk also depend on the levels of risk in the host countries. These levels were similar in all 3 host countries for breast, prostate, and intestinal cancer, whereas they differed markedly for oesophagus, stomach and lung cancer.

Considering the effects of time trends, of the differences in migrant selection, and of the dissimilarities of some of the risk levels prevailing in the host countries, it is not surprising that the present findings differ in some respects from the results of the earlier studies on cancer among Polish migrants.

*Stomach* cancer mortality rates (higher in Poland than in England and Wales and intermediate in the migrants) among migrants to England and Wales appear to be closer to the rates in the host country than was observed in the United States, particularly in 1950, and Australia. This may be due, at least in part, to the higher incidence of this cancer in England and

FIG. 8.—Male and female age-specific mortality rates from lymphomas in England and Wales (x x x) and among Polish migrants to England and Wales in 1970–72 (○- - -○) and in Poland in 1971 (x——x).

Wales than in the other two host countries. Selective migration factors have probably also had some bearing, migrants to England and Wales being more from the lower-risk groups, the higher socio-economic classes.

*Intestinal-tract* cancer mortality rates for Polish female migrants dwelling in all 3 host countries approached the same high level experienced by their native populations, whereas for males this occurred only in the United States. As the migrants to the United States originated more from rural areas where the risk of this cancer was probably the lowest, one would expect their transition to the high risk to be, if anything, slower than for migrants to England and Wales and Australia derived from higher risk groups. The different responses for this cancer between males and females is hard to explain. The deficit among Polish migrants to England and Wales in the 60–64 age group is probably due to random variation; but it is observed in both sexes, and in cancers of breast (female) and prostate (male), neoplasms known to have many epidemiological features common with intestinal-tract cancer.

The increase in *breast*-cancer risk from the low levels in Poland to close to the higher levels in all 3 host countries is noteworthy. This transition was virtually complete in England and Wales and in Aus-

FIG. 9.—Male age-specific mortality rates from leukaemias in England and Wales (x x x) and among Polish migrants to England and Wales in 1970–72 (O- - -O) and in Poland in 1971 (x——x).

FIG. 10.—Truncated age-adjusted cancer mortality ratios for ages 40–79 by site and sex in England and Wales and among Polish migrants to England and Wales in 1970–72 and in Poland in 1971. (For methods of adjustment see text.) The number of deaths among migrants aged 40–79 is given in parenthesis at the right hand margin of the respective bars. Note that these are not the same as in Table I which pertain to 35–85+.

tralia, whereas in the United States in 1950 the increased rate affected mainly the younger age groups and the overall rate was still only 68% of that for native white Americans, whereas by 1959–61 it had risen to 85% of that rate. A similar trend was observed for *prostatic* cancer, but comparisons are based on smaller numbers and on less reliable data, as this cancer occurs at an age when reliability of comparison of cancer statistics is at its lowest.

Whereas in the United States overall male *lung*-cancer mortality was distinctly higher among the Polish-born than among native Americans, Polish migrants to England and Wales experienced a mortality clearly lower than that in the population of their country of adoption. This difference, least distinct in the oldest cohorts, is partly related to the very high level of lung-cancer mortality in England and Wales—much higher than in the United States or in Poland. Among Polish male migrants born before 1895–1900, lung-cancer mortality was higher than among the natives in each of the 3 host countries, this difference being least apparent for England and Wales, where the rates were anyway high. Probably most of these older migrants left Poland before the Second World War, were born in rural farming areas and settled, particularly in the United States, in urban areas. It is interesting to note that an increased risk of lung cancer, independent of smoking history, in migrants from rural farming to urban areas has been described for the U.S. native whites by Haenszel *et al.* (1962)

Female lung-cancer mortality was higher in all the Polish migrant groups discussed than in Poland, being similar to levels in the host country in England and Wales,* whereas the levels were higher than those in the native-born in the U.S. In both sexes this difference diminished, but did not disappear when comparison was limited to U.S. metropolitan areas.

The relatively high *leukaemia* mortality of Polish-born male migrants to England and Wales reflects the higher risk in their country of origin. This explanation does not hold for *lymphomas*, mortality from which was significantly increased among these migrants, although low in Poland. A less consistent increase in leukaemia and lymphoma risk was found among Polish-born Americans, but in 1950 for both these cancers (both sexes) they ranked second or third highest, after migrants from the U.S.S.R., among the United States foreign-born (Haenszel, 1961). The high incidence of these neoplasms in Jews is known and about 17% of Polish-born Americans reported Yiddish as their mother tongue (Haenszel, 1961; U.S. Census of Population, 1960); no similar data are available for England and Wales, but the percentage of Jews would probably be lower among Polish migrants there, and the increase in risk, particularly for lymphomas, among Polish migrants to England and Wales seems too large to be accounted for by the Jewish fraction.

From the data presented, it is impossible to tell to what extent the differences in risk between the various populations of Polish migrants are the effect of different environmental characteristics of their place of destination, of differing length of their residence there, or of different environment before leaving Poland. The effect of changes with time in cancer risk is also difficult to evaluate, but the follow-up of these migrants can provide information on these trends. As to the other factors, special studies of the habits, customs and environment of the migrants are required. The increasing importance of breast and prostate as well as intestinal-tract cancers indicates that priority should be given to studies covering characteristics possibly related to their aetiology.

This work was partly supported by the PL 480 Agreement 05-009-01 sponsored by the National Cancer Institute, Bethesda, Maryland 20014, U.S.A.

### REFERENCES

DOLL, R. & COOK, P. (1967) Summarizing indices for comparison of cancer incidence data. *Int. J. Cancer*. **2**, 269.

HAENSZEL, W. (1961) Cancer mortality among the foreign-born in the United States. *J. Natl Cancer Inst.*, **26**, 37.

HAENSZEL, W., LOVELAND, D. B. & SIRKEN, M. G. (1962) Lung cancer mortality as related to residence and smoking histories. I. White males. *J. Natl Cancer Inst.*, **28**, 947.

STASZEWSKI, J. (1974) Cancer of the upper alimentary tract and larynx in Poland and in Polish-born Americans. *Br. J. Cancer*, **29**, 389.

STASZEWSKI, J. (1976) *Epidemiology of Cancer of Selected Sites in Poland and Polish Migrants.* Cambridge, Mass.: Ballinger Publ. Co.

STASZEWSKI, J. & HAENSZEL, W. (1965) Cancer mortality among the Polish-born in the United States. *J. Natl Cancer Inst.*, **35**, 291.

STASZEWSKI, J., MCCALL, M. & STENHOUSE, N S. (1971) Cancer mortality in 1962–1966 among Polish migrants to Australia. *Br. J. Cancer*, **25**, 599.

STASZEWSKI, J., MUIR, C. S., SLOMSKA, J. & JAIN, K. (1970) Sources of demographic data on migrant groups for epidemiological studies of chronic diseases. *J. Chron. Dis.*, **23**, 351.

U.S. CENSUS OF POPULATION (1960) *Mother Tongue of the Foreign Born.* Washington, D.C.: U.S. Department of Commerce, Bureau of the Census.

* Although there appears to be a difference in the age-standardized rates in Fig. 10, for all age groups combined there were 40 female lung-cancer deaths among those migrants, 38·9 deaths being expected.